# The protective effects of baicalin for respiratory diseases: an update and future perspectives

**DOI:** 10.3389/fphar.2023.1129817

**Published:** 2023-03-16

**Authors:** Siyu Song, Lu Ding, Guangwen Liu, Tian Chen, Meiru Zhao, Xueyan Li, Min Li, Hongyu Qi, Jinjin Chen, Ziyuan Wang, Ying Wang, Jing Ma, Qi Wang, Xiangyan Li, Zeyu Wang

**Affiliations:** ^1^ Key Laboratory of Active Substances and Biological Mechanisms of Ginseng Efficacy, Jilin Provincial Key Laboratory of Bio-Macromolecules of Chinese Medicine, Ministry of Education, Northeast Asia Research Institute of Traditional Chinese Medicine, Changchun University of Chinese Medicine, Changchun, Jilin, China; ^2^ GCP Department, Affiliated Hospital of Changchun University of Chinese Medicine, Changchun, China; ^3^ College of Integrated Traditional Chinese and Western Medicine, Changchun University of Chinese Medicine, Changchun, China; ^4^ College of Traditional Chinese Medicine, Changchun University of Chinese Medicine, Changchun, China

**Keywords:** baicalin, respiratory diseases, molecular mechanisms, pharmacological action, pharmacokinetics, baicalin-loaded nano-delivery system

## Abstract

**Background:** Respiratory diseases are common and frequent diseases. Due to the high pathogenicity and side effects of respiratory diseases, the discovery of new strategies for drug treatment is a hot area of research. *Scutellaria baicalensis* Georgi (SBG) has been used as a medicinal herb in China for over 2000 years. Baicalin (BA) is a flavonoid active ingredient extracted from SBG that BA has been found to exert various pharmacological effects against respiratory diseases. However, there is no comprehensive review of the mechanism of the effects of BA in treating respiratory diseases. This review aims to summarize the current pharmacokinetics of BA, baicalin-loaded nano-delivery system, and its molecular mechanisms and therapeutical effects for treating respiratory diseases.

**Method:** This review reviewed databases such as PubMed, NCBI, and Web of Science from their inception to 13 December 2022, in which literature was related to “baicalin”, “*Scutellaria baicalensis* Georgi”, “COVID-19”, “acute lung injury”, “pulmonary arterial hypertension”, “asthma”, “chronic obstructive pulmonary disease”, “pulmonary fibrosis”, “lung cancer”, “pharmacokinetics”, “liposomes”, “nano-emulsions”, “micelles”, “phospholipid complexes”, “solid dispersions”, “inclusion complexes”, and other terms.

**Result:** The pharmacokinetics of BA involves mainly gastrointestinal hydrolysis, the enteroglycoside cycle, multiple metabolic pathways, and excretion in bile and urine. Due to the poor bioavailability and solubility of BA, liposomes, nano-emulsions, micelles, phospholipid complexes, solid dispersions, and inclusion complexes of BA have been developed to improve its bioavailability, lung targeting, and solubility. BA exerts potent effects mainly by mediating upstream oxidative stress, inflammation, apoptosis, and immune response pathways. It regulates are the NF-κB, PI3K/AKT, TGF-β/Smad, Nrf2/HO-1, and ERK/GSK3β pathways.

**Conclusion:** This review presents comprehensive information on BA about pharmacokinetics, baicalin-loaded nano-delivery system, and its therapeutic effects and potential pharmacological mechanisms in respiratory diseases. The available studies suggest that BA has excellent possible treatment of respiratory diseases and is worthy of further investigation and development.

## 1 Introduction

Respiratory diseases are a significant worldwide health problem with high morbidity and mortality rates. Smoking, radiation, dust, bacteria, viruses, *mycoplasma*, and obesity can all cause pulmonary diseases, which are coronary viral disease-2019 (COVID-19), acute lung injury (ALI), asthma, chronic obstructive pulmonary disease (COPD), pulmonary arterial hypertension (PAH), pulmonary fibrosis (PF), and lung cancer (LC) (Venkatesan et al., 2007; Kumar et al., 2018). Due to its susceptibility and poor prognosis, drug development in this field has been of great interest.

Chinese herbal medicine, as one of the natural products, has been a rich source of discovery compounds (Hu et al., 2021). *Scutellaria baicalensis* Georgi (SBG) is a *Lamiaceae* family herb widely used in East Asian countries to treat diseases (Shang et al., 2010). Baicalin (BA), baicalein (BE), wogonin, and wogonoside are the main flavonoid compounds found in SBG (Pan et al., 2021). As the main effective constituent, BA has been found to have therapeutic effects on cardiovascular, hepatobiliary, brain, nervous system, and intestinal diseases (Jin et al., 2019; Song et al., 2020; Xin et al., 2020; Ganguly et al., 2022). BA has been extensively reported to treat respiratory diseases (Deng et al., 2022; Ju et al., 2022; Li et al., 2022). However, the widespread use of BA is still a challenge due to its low solubility and poor bioavailability (Haider et al., 2018; Pramual et al., 2022). And the molecular mechanism of BA in treating respiratory diseases has yet to be reported based on a summary of reliable evidence.

Herein, we discuss a comprehensive report of BA on pharmacokinetics and baicalin-loaded nano-delivery system. In addition, we have summarized studies related to BA for the treatment of COVID-19, ALI, asthma, PAH, COPD, PF, and LC. And the main therapeutic targets and the critical signal pathways of BA against respiratory diseases were summarized. This review could provide a theoretical basis for further pharmacological studies and clinical applications of BA for the prevention and treatment of respiratory diseases.

## 2 Literature survey databases

This review reviewed databases such as PubMed, NCBI, and Web of Science from their inception to 13 December 2022, using terms including “baicalin”, “*Scutellaria baicalensis* Georgi”, “COVID-19”, “acute lung injury”, “pulmonary arterial hypertension”, “asthma”, “chronic obstructive pulmonary disease”, “pulmonary fibrosis”, “lung cancer”, “pharmacokinetics”, “liposomes”, “nano-emulsions”, “micelles”, “phospholipid complexes”, “solid dispersions”, “inclusion complexes”, and other terms. The pharmacokinetics of BA, strategies to improve bioavailability, therapeutic effects, and potential pharmacological mechanisms were summarized. Further research in this area is informed and recommended.

## 3 Pharmacokinetics of BA

As a glycoside flavonoid, BA has low aqueous solubility and poor membrane permeability, reducing its oral bioavailability and limiting its clinical application (Taiming and Xuehua, 2006; Ju et al., 2022). *In vivo*, BA is rapidly hydrolyzed to its glycosidic ligand BE, which shows excellent permeability and lipophilicity (Wang et al., 2020a). Liu *et al.* investigated the absorption mechanisms of BA and BE in rats and discovered that BA was moderately absorbed in the stomach and poorly absorbed in the small intestine and colon. While BE was well absorbed in the stomach and small intestine and relatively little in the colon (Taiming and Xuehua, 2006). After intravenous BA administration, the drug concentration decreases from high to low in the major organ tissues in the following order: kidney, lung, liver, and spleen (Wei et al., 2016). Notably, BA is the main metabolite in the blood, whether an oral BA or BE (Akao et al., 2013).

The metabolism of BA is a crucial factor in determining its efficacy and toxicity. The first step in the metabolism of BA is the hydrolysis of the glycoside into its ligand BE by the enzyme β-glucuronidase produced by intestinal microbiota (Xing et al., 2005). The type and abundance of intestinal microbes can impact the metabolic processes of BA in the intestine, making the intestinal microbiota an important factor in the metabolic processes of BA (Xie et al., 2020). This was confirmed by conducting studies in a rat model of antibiotic-pretreated rats, which showed that the maximum serum concentration (C_max_), terminal half-life, elimination rate constant, and plasma drug concentration-time curve (AUC) values were significantly altered compared to normal rats. Moreover, gut microbiota, such as *Escherichia coli* and *Streptococcus* spp., can produce β-glucuronidase which enhances the metabolism of BA (Ishihara et al., 2002).

After metabolism in the intestine, BA undergoes glucuronidation (Zeng et al., 2022). Extensive hepatic and intestinal first-pass glucuronidation of BA has been found in both humans and rats (Akao et al., 2000; Zhang et al., 2007). BA and its glycosidic ligand exhibit inhibitory effects in the liver on UDP-glucuronosyltransferase (UGT) isoforms, including UGT1A1, 1A6, 1A9, and 2B7 (Teng et al., 2013). As one of the essential phase II mechanisms, those UGT isoforms might affect the bioavailability of BA.

Following glucuronidation, the hepatic-intestinal circulation of BA is comprised of several key steps, including uptake by the liver from the blood, excretion from the liver into the bile, transport of the bile to the duodenum for reabsorption, and finally return to the liver *via* the portal circulation (Xing et al., 2005). During this process, BA has two potential two sites of absorption. The first site is the upper intestine, which may directly absorb BA, and the second site is located in the colon in the form of glycosylated ligands (Lu et al., 2007).

As with the intestine, extensive metabolism of BA occurs in the liver. After oral administration of BA to rats, 32 metabolites were detected in blood and urine. During this process, various reactions were found in rats, including methylation, hydrolysis, hydroxylation, methoxylation, glucuronide conjugation, sulfate conjugation, and complex reactions. The liver and kidney are the most important organs for metabolite distribution (Zhang et al., 2015). Finally, BA is excreted mainly as gluconate in the bile, accompanied by a small amount of urine (Xing et al., 2005).

In the intestine, β-glucuronidase, which is produced by the intestinal flora, hydrolyzes BA to BE. Glucuronidation is a significant metabolic pathway for BA, which undergoes various reactions in the liver and kidneys, the main metabolic organs. The elimination of metabolites occurs primarily through bile and urine. The biotransformation of BA requires the metabolic enzymes UGT and β-glucuronidase. The pharmacokinetics of BA can also be affected by various factors, including the administration route. The study by (Xing et al., 2005) found that the AUC values for BA administered intravenously (37 μmol/kg) and orally (224 μmol/kg) were 33.57 ± 1.8 (nmol·h/mL) and 4.43 ± 0.4 (nmol·h/mL), respectively. This indicates that the intravenous administration of BA was 6.05 times lower in dose but 7.57 times higher in AUC compared to the oral administration. However, BA’s low bioavailability limits its clinical application, leading to the development of various formulations to enhance its solubility, bioavailability, and lung targeting potential.

## 4 Baicalin-loaded nano-delivery system

In recent years, the combination of nanoscience and biologically active natural compounds has been frequently investigated to create safe, biodegradable, and biocompatible drug delivery systems. Poor bioavailability is the main problem limiting BA in treating respiratory diseases (Xu et al., 2022a). In recent years, the development of new formulations for BA has been of increasing interest to the pharmaceutical field. Various nano-sized delivery systems have been explored, including liposomes, nano-emulsions, micelles, phospholipid complexes, solid dispersions, and inclusion complexes (Li J. et al., 2009; Li et al., 2013; Yue et al., 2013; Zhao et al., 2013; Wu et al., 2014; Zhang et al., 2014) (Tables 1, 2).

**TABLE 1:** Baicalin delivery systems and improved properties.

Delivery system	Improved properties	Refs.
Liposomes	Sustained drug release; increased oral availability, the peak concentration, and the drug concentrations in the liver, kidney, and lung	Wei et al. (2014)
Liposomes	Prolonged the duration time *in vivo*; increased the drug concentration of lung; inhibited LPS-induced inflammation in mice	Long et al. (2020)
Liposomes	Inhibited the growth rate of nude mice bearing orthotopic human lung cancer; improved lung targeting; sustained drug release; increased drug concentration in the lungs	Wei et al. (2017)
Liposomes	Improved cytotoxicity and cellular uptake; sustained drug release	Chen et al. (2016)
Nano-emulsions	Enhanced drug solubility and stability; prolonged the duration time *in vivo*	Wu et al. (2018)
Nano-emulsions	Improved oral bioavailability; sustained drug release	Zhao et al. (2013)
Micelles	Sustained drug release; improved solubility	Zhang et al. (2014)
Micelles	Improved cellular uptake and cytotoxicity; improved targeting of tumors; reduced side effects and growth of tumor volume in A549 tumor-bearing nude mice	Wang et al. (2019)
Phospholipid complexes	Improved the solubility, biomembrane permeation, and bioavailability	Wu et al. (2014)
Solid dispersions	Improved the dissolution and bioavailability	Li et al. (2013)
Solid dispersions	Enhanced the dissolution rate and bioavailability	Cui et al. (2016)
Inclusion complex	Improved aqueous solubility, dissolution rate, and oral bioavailability	Li et al. (2017)
Inclusion complex	Increased solubility, stability, and antioxidant activity	Li et al. (2009a)

Refs., references; LPS, lipopolysaccharide.

**TABLE 2:** Drug delivery systems for baicalin studied in preclinical acute lung injury and lung cancer models.

Disease	DDSs	Carrier	Experimental model	Loading efficiency	Encapsulation efficiency	Main results	Refs.
ALI	Liposome	Cholesterol/soy lecithin	Mice	NA	NA	*In vivo*: BA liposome had a better effect on reducing the wet/dry ratio, alleviating the lung injury score, and decreasing the proinflammatory factors (TNF-α and IL-1β) and total proteins in BALF	Long et al. (2020)
LC	Liposome	HSPC/Tween-80/citric acid/BA	A549 cells/mice/rats	NA	82.8% ± 1.24%	*In vivo*: BA-loaded nanoliposomes showed better antitumor therapeutic efficacy in the nude mice bearing orthotopic human lung cancer	Wei et al. (2017)
LC	Micelles	Que/S-S/oHA/Man/FA	A549 cells/RAW264.7 cells/mice	3.50 ± 0.34%	72.63% ± 7.1%	*In vivo*: effective antitumor activity and reduced side effects of micelles were confirmed through antitumor experiments in A549 tumor-bearing nude mice	Wang et al. (2019)
						*In vitro*: good cellular penetration and tumor cytotoxicity of micelles were demonstrated through cellular studies	

DDSs, drug delivery systems; HSPC, phosopholipon 90H; BA, baicalin; NA, not applicable; BALF, bronchoalveolar lavage fluids; Que/S-S/oHA/Man/FA, quercetin/dithiodipropionic acid/oligomeric hyaluronic acid/mannose/ferulic acid; TNF-α, tumor necrosis factor-alpha; IL, interleukin; Refs., references; ALI, acute lung injury; LC, lung cancer.

### 4.1 Liposomes

Liposomes are the most widely studied nano drug delivery system due to their synergistic delivery system that increases drug solubility (Huang et al., 2022; Ramedani et al., 2022). It has been demonstrated that the advantage of BA-loaded nanoliposomes for treating respiratory diseases is lung targeting (Wei et al., 2016; Wei et al., 2017). Research has shown that the BA liposome (L-BA) improves oral availability and tissue distribution (Wei et al., 2014). Compared to the same dose of BA, L-BA (100 mg/kg, tail-vein injection) more significantly reduced the W/D ratio, lung injury score, pro-inflammatory factors, and protein in total bronchoalveolar lavage fluid in a mouse model of lipopolysaccharide (LPS)-induced ALI (Long et al., 2020). In particular, the daily increase in tumor weight of BALB/c nude mice injected with A549 human lung cancer tumor cells was significantly reduced by L-BA (100 mg/kg) after intravenous injection of the drug, resulting in increased the survival rate of mice (Wei et al., 2017). L-BA was modified with folic acid and polyethylene glycol to improve further tumor targeting, which exhibited higher cellular uptake than non-targeted liposomes (Chen et al., 2016).

### 4.2 Nano-emulsions

Nano-emulsions offer unique advantages and properties that increase water solubility and dynamic stability, improve therapeutic efficacy, and reduce adverse effects and toxic reactions (Pangeni et al., 2018). Due to the sustained-release characteristics of the nano-emulsions containing BA, it is very effective in increasing oral availability. A novel nano-emulsion has been proven to improve systemic exposure to BA by promoting intestinal absorption and lymphatic transport (Wu et al., 2018). Zhao et al. (2013) prepared BA-loaded nano-emulsions by internal and external drug addition. And they found that both were superior to BA suspensions in terms of C_max_ and AUC_0-∞_.

### 4.3 Micelles

Micelles are synthesized from amphiphilic surfactants in the aqueous phase, which provides hydrophobic cores as reservoirs to increase the solubility of water-insoluble drugs (Gaucher et al., 2005). The carrier material containing a mixture of Pluronic P123 copolymer and sodium taurocholate in bundle gum increased the C_max_ and AUC in rats by 1.77 and 1.54 times more than the BA suspension, respectively (Zhang et al., 2016). The therapeutic effects of BA in micelles have been investigated in preclinical LC models. Novel carrier materials have been screened for structures targeting nano-micelles (named “nano-dandelion”) for the simultaneous delivery of curcumin and BA. The nano-dandelion improved cellular uptake and cytotoxicity in the A549 cells, exhibiting better anti-cancer effects. In addition, by aggregating more readily at the tumor site, tail vein injection nano-dandelion exerts a more effective tumor suppression effect than free drug (Wang et al., 2019).

### 4.4 Phospholipid complexes

As with liposomes, phospholipid complexes can improve the affinity of BA to solubility and cell membranes (Qin et al., 2018). It has been shown that orally administered SBG extract-phospholipid complex improves solubility and bioavailability *in vivo* (Chen S. et al., 2022). Although phospholipid complexes can improve bioavailability, the increased lipophilicity leads to poor solubility and dispersion of the drug in the aqueous phase (Huang et al., 2019; Wang et al., 2020b). Self-emulsifying drug delivery systems (SEDDS) are isotropic mixtures of drugs, lipids, and surfactants, which are well-suited for lipophilic drugs with limited solubility (Salawi, 2022). A preparation for improving the oral availability of BA has been developed by combining a phospholipid complex with a SEDDS, which has been shown to enhance BA transport and relative bioavailability (Wu et al., 2014).

### 4.5 Solid dispersions

Solid dispersions improve the bioavailability of drugs by reducing the particle size to an absolute minimum, thereby increasing the wettability of the drug (Vasconcelos et al., 2007). Li et al. (2013) have developed that the BA-polyvinylpyrrolidone coprecipitate was prepared by the solid phase dispersion technique solvent method. Compared to the BA raw material capsules, the relative bioavailability of the coprecipitate capsules was 338.2% ± 93.2%. BA mesoporous carbon nanopowder (MCN) solid dispersions offer more advantages over pure BA as an oral delivery system, including shorter time to peak concentration and higher C_max_ and AUC. Significantly, it improves solubility and oral bioavailability without gastrointestinal and renal toxicity (Cui et al., 2016).

### 4.6 Inclusion complexes

Cyclodextrins have been widely used as functional excipients in inclusion formulations (Gandhi et al., 2020). Li et al. (2017) synthesized β-cyclodextrin (β-CD) and BA host-guest inclusion complexes to exert better therapeutic effects. And the inclusion complex found that the AUC_0-t,_ AUC_0-∞_, and the relative oral bioavailability increased by 2.65, 2.53, and 2 times compared with free BA. To further improve the solubility and stability of the complexation of BA with β-CD, researchers structurally modified the BA complex to synthesize hydroxypropyl-β-CD to enhance antioxidant capacity, which is the most reactive form (Li et al., 2009a).

Due to the low bioavailability of BA, researchers have synthesized various novel formulations of BA to increase its solubility, bioavailability, targeting, cellular uptake, and retention time *in vivo*, increasing the therapeutic effect of BA. However, studies of these novel formulation forms are still in the pre-clinical stage, and further evaluation is needed to determine whether they can be used in the clinic.

## 5 The protection of BA against respiratory diseases

### 5.1 COVID-19 prevention

In 2019, the unprecedented COVID-19 was caused by the new coronavirus severe acute respiratory syndrome coronavirus 2 (SARS-CoV-2), which has become a global public health concern (Kciuk et al., 2022; Murali et al., 2022). BA has been shown to inhibit SARS-CoV-2 RNA-dependent RNA polymerase activity and exhibited significant antiviral activity against SARS-CoV-2 *in vitro* (Zandi et al., 2021). Evidence shows that cytokine storm (CS) was a pivotal stage in the advancement of COVID-19 (Hach et al., 2022; Jing et al., 2022; Shevtsova et al., 2022). You et al. (2022) used the method of network pharmacology to analyze the possible pathway of BA inhibiting CS. The enrichment analysis results showed that the tumor necrosis factor-α (TNF-α) pathway and IL (interleukin)-17 pathway might be potentially significant pathways for BA to inhibit CS. Furthermore, BA (200 mg/kg, gavage) exerts an anti-inflammatory effect by decreasing the expression of TNF-α, which alleviates CS against lung injury *in vivo*. As is well known, SARS-CoV-2 uses the ACE2 to enter cells, and ACE2 cleaves angiotensin (Ang) II into Ang 1–7, which is thought to exert a cellular response primarily through the receptor Mas to counteract RAS activation in several organs (Letko et al., 2020; South et al., 2020). A study by Wei reported that BA inhibits Ang II-induced oxidative damage and dysfunction in endothelial cells through activation of the ACE2/Ang-(1–7)/Mas axis and upregulation of the PI3K/AKT/eNOS pathway, thereby exerting a protective effect on endothelial cells (Wei et al., 2015).

**FIGURE 1:**
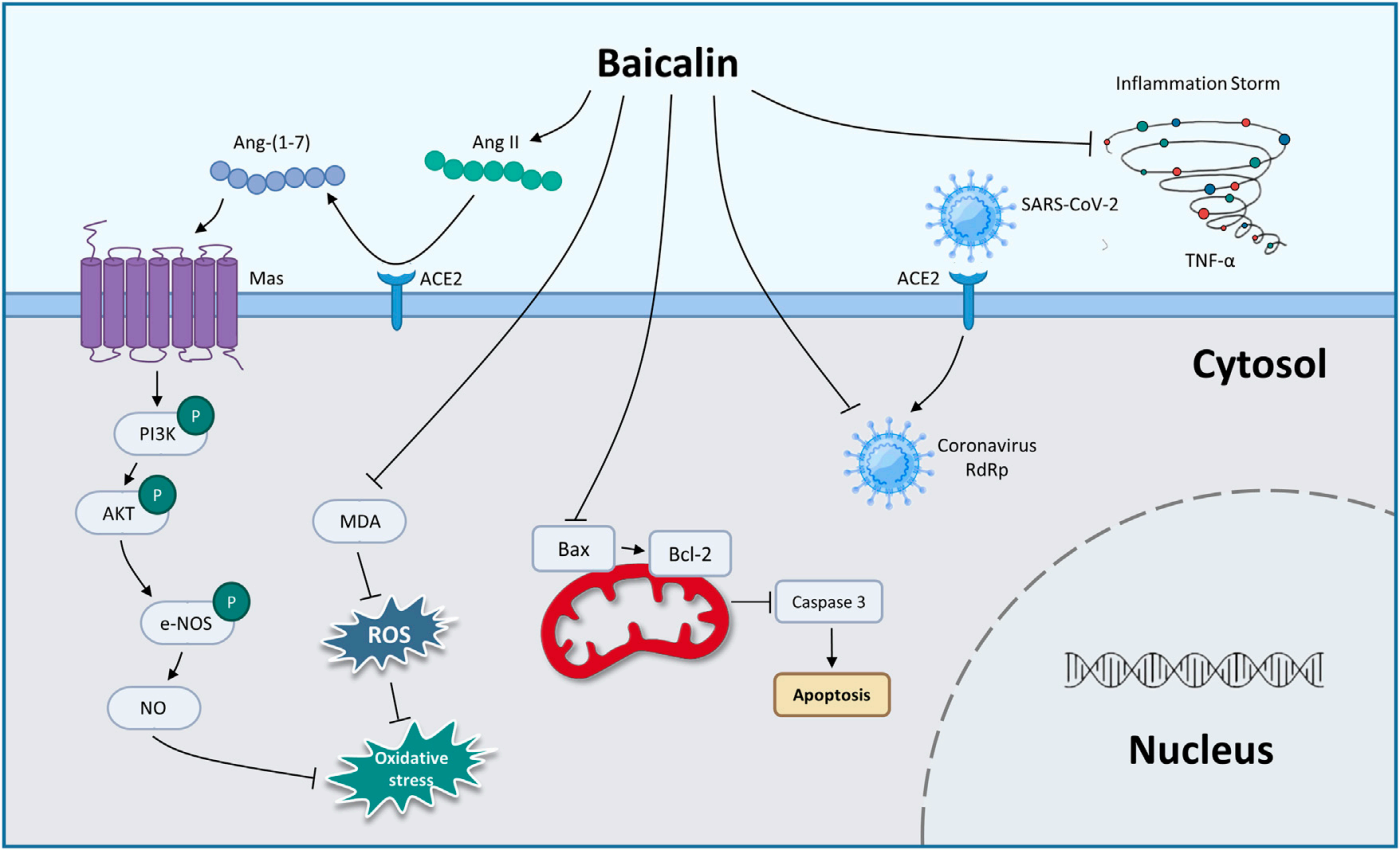
The therapeutic mechanism of baicalin on coronary viral disease-2019 (graphics courtesy of freepik.com).

From the above summary, we found that BA prevented COVID-19 through antiviral activity, inhibiting CS and protecting against endothelial cell dysfunction and oxidative damage (Figure 1; Table 3). In the future, clinical evaluation of BA for the treatment of COVID-19 needs to be performed.

**TABLE 3:** Summary of the targets/pathways/mechanisms and effects of baicalin on coronary viral disease 2019 and acute lung injury.

Disease	Inducer	Experimental model	Dose	Targets/pathways/mechanisms	Effects	Refs.
COVID-19	*In vitro*: SARS-CoV-2 (0.1 MOI)	*In vitro*: the Vero CCL-81 cells	*In vitro*: 1.1–30 μM	*In vitro*: SARS-CoV-2 RdRp↓	Inhibits SARS-CoV-2 RdRp	Zandi et al. (2021)
		Human Calu-3 cells				
COVID-19	*In vivo*: LPS (5 mg/kg)	*In vivo*: male C57BL/6J mice	*In vivo*: 200 mg/kg (gavage)	*In vivo*: TNF-α, total cells in BALF↓	Inhibits cytokine storm *via* TNF-α and IL-17 pathway	You et al. (2022)
	*In vitro*: LPS (100 ng/mL)	*In vitro*: MH-S cells	*In vitro*: 12.5–100 μg/mL	*In vitro*: TNF-α↓		
COVID-19	*In vitro*: Ang II (1 × 10^-6^mol/L)	*In vitro*: HUVECs cells	*In vitro*: 12.5–50 μmol/L	*In vitro*: Bcl-2, Ang-(1–7), ACE2 activity, NO, T-AOC, PI3K, p-AKT, p-eNOS/eNOS↑	Protects endothelial dysfunction and oxidative stress *via* modulating the expression of Bax, Bcl-2, and cleaved caspase-3, activating ACE2/Ang-(1–7)/Mas axis and up-regulating PI3K/AKT/eNOS pathway	Wei et al. (2015)
				Bax, cleaved caspase-3, Ang II, MDA, ROS↓		
				mRNA and protein expression of ACE2 and Mas↑		
ALI	*In vivo*: LPS (3 mg/kg, i.t. administration)	*In vivo*: SPF male mice	*In vivo*: 200 mg/kg (orally administration)	*In vivo*: MDA, TNF-α, IL-1β, IL-6, total leukocyte counts, neutrophil counts, macrophage counts, lymphocyte counts↓	Enhances antioxidant systems and significantly and reduces both inflammatory cells and mediators *via* the Nrf2-mediated HO-1 signaling pathway	Meng et al. (2019)
				Nrf2, HO-1, CAT, SOD↑		
ALI	*In vivo*: LPS 10 mg/mL (100 μl) by airway instillation	*In vivo*: male Wistar rats	*In vivo*: 50 and 100 mg/kg (gavage)	*In vivo*: protein concentration in BALF, number of neutrophils, TNF-α, IL-1β, IL-6, CXCL1, MPO, TLR4, MyD88, NLRP3, p-NF-κB/NF-κB, p-ERK/ERK, p-p38 MAPK/p38 MAPK↓	Reduces the permeability of the alveolocapillary membrane, alleviates tissue injury and inflammatory infiltration, and inhibits the secretion of inflammatory factors and the infiltration of neutrophils *via* TLR4/MyD88/NF-κB/NLRP3 and MAPK pathway	Changle et al. (2022)
	*In vitro*: LPS 50 μg/mL	*In vitro*: BEAS-2B cells	*In vitro*: 5 and 10 μg/mL	*In vitro*: IL-8, IFN-γ, TNF-α, IL-1β, IL-6, GM-CSF, p-NF-κB/NF-κB, NLRP3↓		
				mRNA and protein expression of TLR4 and MyD88↓		
				mRNA and protein expression of NF-κB↑		
ALI	*In vivo*: APEC-O78 strain (2 × 10^9^ CFU) by i.t. inoculation	*In vivo*: male Hyline Brown healthy chickens	*In vivo*: 50, 100, and 200 mg/kg by oral gavage	*In vivo*: TNF-α, IL-1β, IL-6, MPO, p-IκB, p-p65↓	Reduces the W/D ratio, MPO activity, and production of IL-1β, TNF-α, and IL-6 of lung tissues by regulating NF-κB signaling pathway	Peng et al. (2019)
ALI	*In situ*: infusion of air (0.25 mL/min for 1 min)	*In situ*: isolates and perfuses lungs *in situ* of male SD rats	*In situ*: 1, 2, and 4 mg/kg	*In situ*: protein concentration in BALF, CINC-1, TNF-α, NF-κB, MPO, MDA↓	Reduce the production of proinflammatory cytokines, oxygen radicals, and NF-κB activity	Li et al. (2009b)
				IκB-α↑		

COVID-19, coronary viral disease-2019; TNF-α, tumor necrosis factor-alpha; BALF, bronchoalveolar lavage fluid; IL, interleukin; SARS-CoV-2, Severe Acute Respiratory Syndrome Coronavirus 2; RdRp, RNA-Dependent-RNA, polymerase; Ang, angiogenin; Bcl-2, B-cell lymphoma-2; ACE2, Angiotensin-converting enzyme 2; NO, nitric oxide; T-AOC, total antioxidant capacity; PI3K, Phosphatidylinositol3-kinase; AKT, protein kinase B; NOS, nitric oxide synthase; Bax, Bcl-2-associated X protein; MDA, malondialdehyde; ROS, reactive oxygen species; Nrf2, nuclear factor erythroid 2-related factor 2; HO-1, heme oxygenase-1; CAT, catalase; SOD, superoxide dismutase; CXCL1, chemokine 1; MPO, myeloperoxidase; TLR4, Toll-like receptor 4; MyD88, myeloid differentiation factor 88; NLRP3, NOD-like receptor family pyrin domain containing 3; NF-κB, nuclear factor-kappaB; ERK, extracellular signal-regulated kinase; MAPK, Mitogen-activated protein kinase; IFN-γ, interferon-gamma; GM-CSF, granulocyte-macrophage colony-stimulating factor; CINC-1, cytokine-induced neutrophil chemoattractant-1; IκB-α, Inhibitor of kappaB-α; SD, Sprague-Dawley; LPS, lipopolysaccharide; i.g., intragastric gavage; SPF, specific pathogen-free; APEC, avian pathogenic *Escherichia coli*; i.n., intranasal; i.t., intratracheal; HUVECs, Human umbilical vein endothelial cells; Refs., references; ALI, acute lung injury.

### 5.2 Acute lung injury

ALI is a life-threatening disease with a high fatality rate in a clinical setting (Guo et al., 2022). Severe lung infection, pulmonary contusion, and pulmonary embolism are the direct causes of ALI (He et al., 2021). The main pathological features are acute inflammation and apoptosis of alveolar epithelial cells (Allen and Kurdowska, 2014; Liu et al., 2020; Gao et al., 2021).

Using the LPS-induced ALI model, BA might be a novel strategy for lung protection, mainly due to BA (200 mg/kg, orally administration) inhibited the expression of TNF-α, IL-1β, IL-6, and malondialdehyde (MDA) and restored antioxidative enzyme activities, including superoxide dismutase (SOD) and catalase (CAT). The suppression of inflammatory and oxidant responses was mediated by activation of the nuclear erythroid factor 2 (Nrf2)-mediated heme oxygenase-1 (HO-1) pathway (Meng et al., 2019). In addition, another similar model study conducted by Zhu *et al.* demonstrated that 50 mg/kg and 100 mg/kg of BA (gavage) significantly alleviated the permeability of the alveolocapillary membrane and tissue injury through the toll-like receptor 4 (TLR4)/myeloid differentiation factor 88 (MyD88)/nuclear factor-kappa B (NF-κB)/nod-like receptor pyrin containing 3 (NLRP3) pathway and the mitogen-activated protein kinase (MAPK) pathway (Changle et al., 2022). The BA-induced attenuation of lung injury *via* the NF-κB anti-inflammatory pathway was confirmed in another lung injury model. Peng showed that BA (80 mg/kg, oral gavage) might alleviate lung injury in an avian pathogenic *E. coli*-induced model by inhibiting the phosphorylation of NF-κB (Peng et al., 2019). The findings demonstrated that BA modulated the NF-κB anti-inflammatory pathway, suggesting that it may contribute to reversing pulmonary injury. In a study of the air embolism-induced ALI model, BA (1, 2, and 4 mg/kg) was added into the lung perfusate, alleviating lung injury in isolated lungs by suppressing the proinflammatory cytokines, oxygen radicals, and NF-κB activity (Li et al., 2009b).

From the above summary, BA exerts anti-oxidative stress and anti-inflammatory effects through the Nrf2-mediated HO-1 pathway. CAT antioxidant enzyme, transforming growth factor-β (TGF-β), and granulocyte-macrophage colony-stimulating factor (GM-CSF) are involved in relieving ALI regulated by BA. And the inhibition of lung inflammation *via* the NF-κB pathway might play a pivotal role in attenuating pulmonary damage by BA (Table 3).

### 5.3 Asthma

Bronchial asthma is a chronic airway disease predominantly characterized by chronic inflammation and hyperresponsiveness, leading to cough, wheezing, shortness of breath, and chest tightness (Olin and Wechsler, 2014; Banno et al., 2020; Yasuda et al., 2020). It is widely believed that cytokines and inflammatory cells such as eosinophils, neutrophils, lymphocytes, and mast cells are involved in asthma (Yasuda et al., 2020; Zhang et al., 2020).

Ma et al. (2014) confirmed that BA (10–40 mg/kg, gavage) alleviated ovalbumin (OVA)-induced allergic asthma by inhibiting airway resistance, eosinophil count, and IL-4 level, recovering lung compliance, and increasing the expression of IFN-γ. And the study of molecular mechanisms might be associated with the decrease of IL-17A. Another similar model also confirmed the importance of immune regulation in asthma. Xu et al. (2017a) demonstrated that intragastric gavage (i.g.) administration of BA at a dose of 10, 25, and 65 mg/kg protected OVA and LPS-induced allergic asthma in mice models by maintaining Th17/Treg balance. In the study of Sun, researchers administered BA (gavage) dosages ranging from 25 to 100 mg/kg/day to mice with OVA-induced allergic asthma. The result demonstrated that BA reduced the expression of TGF-β1, IL-13, and vascular endothelial growth factor in tissue remodeling by suppressing the activation of the extracellular signal-regulated kinase (ERK) pathway and RAS expressions (Sun et al., 2013). Liu et al. (2016) found BA (10, 25, and 50 mg/kg, i.g. administration) could reduce the expression of immunoglobulin E (IgE), IL-6, TNF-α, CC-chemokine receptor (CCR) 7, C-C chemokine ligand (CCL)19/CCL21 in the OVA-induced mice model, inhibiting airway inflammation *via* the NF-κB pathway.

According to above research, immune modulation and inhibition of inflammatory responses appear to be important mechanisms for BA to treat asthma. It is critical for BA to relieve asthma by inhibiting the ERK pathway and inhibiting CCR7, CCL19/CCL21, and NF-κB pathway (Table 4).

**TABLE 4:** Summary of the targets/pathways/mechanisms and effects of baicalin on asthma, chronic obstructive pulmonary disease, and pulmonary arterial hypertension.

Disease	Inducer	Experimental model	Dose	Targets/pathways/mechanisms	Effects	Refs.
Asthma	*In vivo*: OVA (i.p. injection with 10 μg chicken OVA and 2 mg aluminum hydroxide and then inhalation of 1% OVA solution)	*In vivo*: SPF female BALB/c mice	*In vivo*: 10, 20, and 40 mg/kg (gavage)	*In vivo*: WBC, eosinophils, IL-4, IL-17A, IgE↓	Suppresses IL-4, IL-17A, and Th17 cells, improves IFN-γ, and inhibits both the recruitment of eosinophils and mucus overproduction, leading to attenuated airway inflammation and bronchial hyperresponsiveness	Ma et al. (2014)
				Neutrophil, monocytes, lymphocytes→		
				IFN-γ↑		
Asthma	*In vivo*: OVA (100 μg) mixed with LPS (0.1 μg), then 50 μg OVA alone by i.n. instillations	*In vivo*: SPF female BALB/c mice	*In vivo*: 10, 25 and 65 mg/kg (i.g. administration)	*In vivo*: IgE, IL-17A, IL-6, STAT3↓	Protects against allergic asthma by regulating the immunological imbalance of Th17/Treg responses	Xu et al. (2017a)
				IL-10, FOXP3↑		
Asthma	*In vivo*: OVA (100 μg i.p. injection, then inhaled 1% OVA)	*In vivo*: female BALB/c mice	*In vivo*: 25, 50, and 100 mg/kg (gavage)	*In vivo*: TGF-β1, VEGF, IL-13, ERK, p21ras↓	Exerts anti-remodeling effect on asthmatic airway remodeling by decreasing expression of TGF-β1, IL-13, and VEGF and inhibiting the activation of the ERK pathway	Sun et al. (2013)
Asthma	*In vivo*: OVA (20 µg i.p. injection, and then 3% OVA nebulization)	*In vivo*: female BALB/c mice	*In vivo*: 10, 25, and 50 mg/kg (i.g. administration)	*In vivo*: WBC, eosinophils, IgE, CCL19, CCL21, IL-6, TNF-α, p-IκB, p-p65↓	Exerts an inhibitory effect on airway inflammation by inhibiting CCR7 and CCL19/CCL21	Liu et al. (2016)
				mRNA and protein expression of CCR7↓		
COPD	*In vivo*: cigarette smoke (inhalation) and LPS (10 μg, nasal instillation)	*In vivo*: C57BL/6 SPF male mice	*In vivo*: 25, 50, and 100 mg/kg (i.g. administration)	*In vivo*: HSP72↑	Alleviates COPD by upregulating the expression of HSP72 and resulting in the inhibition of JNK signaling activation	Hao et al. (2021)
				Muc5AC, IL-6, IL-8, and TNF-α, cell number, p-JNK/JNK↓		
	*In vitro*: cigarette smoke extract	*In vitro*: MLE-12 cells	*In vitro*: 5, 10, and 20 μmol/L	*In vitro*: HSP72↑, IL-6		
				IL-8, TNF-α, p-JNK/JNK↓		
COPD	*In vivo*: cigarette smoke (inhalation)	*In vivo*: male SD rats	*In vivo*: 40, 80, and 160 mg/kg (i.g. administration)	*In vivo*: IL-1β, TNF-α, p-IκB-α/IκB-α, p-p65/p65↓	Ameliorates airway inflammation by modulating of HDAC2/NF-κB/PAI-1 pathway	Zhang et al. (2021)
				mRNA and protein expression of PAI-1↓		
				HDAC2↑		
	*In vitro*: cigarette smoke extract	*In vitro*: HBE cells	*In vitro*: 10, 20, and 40 μM	*In vitro*: p-IκB-α/IκB-α, p-p65/p65↓		
				HDAC2↑		
				mRNA and protein expression of PAI-1 and TNF-α↓		
COPD	*In vivo*: cigarette smoke (inhalation)	*In vivo*: male SD rats	*In vivo*: 20, 40, and 80 mg/kg (i.g. administration)	*In vivo*: total leukocyte counting, the percentage of neutrophils, nuclear p65, IL-6, IL-8 and TNF-α↓	Exerts anti-inflammatory effect by inhibiting the NF-κB pathway	Lixuan et al. (2010)
				The percentage of mononuclear macrophages and lymphocytes→		
	*In vitro*: cigarette smoke extract	*In vitro*: human type II pneumocytes	*In vitro*: 5, 10, and 20 μmoL			

				*In vitro*: p-p65, IL-6, IL-8 and TNF-α↓		
				IκB-α↑		
PAH	*In vivo*: MCT (60 mg/kg, subcutaneous injection)	*In vivo*: Wistar rats	*In vivo*: 100 mg/kg (i.g. administration)	*In vivo*: mRNA expression of TNF-α, IL-1β, IL-6, and ET-1↓	Exerts protective effects against the lung and heart damage in experimental PAH maybe through inhibiting vascular endothelial inflammatory response	Luan et al. (2015)
				TGF-β1, ICAM-1, NF-κB↓		
				IκB↑		
PAH	*In vivo*: MCT (50 mg/kg, i.p. injection)	*In vivo*: male Wistar rats	*In vivo*: 20, 50, and 200 mg/kg (i.g. administration)	*In vivo*: α-SMA, P/T NF-κB-p65, VCAM, ICAM↓	Protects against experimental PAH *via* regulating the TNF-α/BMPR2 signaling pathway	Xue et al. (2021)
				BMPR2, Smad1/5/8, p-Smad1/5/8, ID1↑		
				mRNA expression of IL-1β and IL-6↓		
				mRNA and protein expression of TNF-α↓		
	*In vitro*: TNF-α (5 ng/mL)	*In vitro*: rat pulmonary artery smooth muscle cells	*In vitro*: 100 μg/mL	*In vitro*: Cyclin D1↓		
				p27, BMPR2, ID1, Smad1/5/8, p-Smad1/5/8↑		
PAH	*In vivo*: MCT (50 mg/kg, i.p. injection)	*In vivo*: male SPF SD rats	*In vivo*: 20, 100, and 200 mg/kg (i.p. injection)	*In vivo*: p-p65/total p65, p-ERK/total ERK↓	Interferes with pulmonary vascular remodeling and PAH development through the AKT/eNOS, ERK, and NF-κB signaling pathways	Yan et al. (2019)
				p-AKT/total AKT, p-eNOS/total eNOS↑		
PAH	*In vivo*: hypoxia	*In vivo*: A_2A_R-deficient Balb/c mice and Balb/c wild-type mice	*In vivo*: 60 mg/kg (i.p. injection)	*In vivo*: CXCR4, SDF-1, P-PI3K/PI3K, P-AKT/AKT↓	Enhances A_2A_R activity and downregulates SDF-1/CXCR4-induced PI3K/AKT pathway	Huang et al. (2017)
				A_2A_R↑		
PAH	*In vivo*: hypoxia	*In vivo*: male SPF SD rats	*In vivo*: 30 mg/kg (i.p. injection)	*In vivo*: mRNA and protein expression of MMP-9↓	Attenuates pulmonary hypertension and cor pulmonale by downregulating the p38 MAPK/MMP-9 pathway	Yan et al. (2016)
				p-p38↓		

OVA, ovalbumin; SPF, specific pathogen free; WBC, white blood cell; IL, interleukin; IgE, Immunoglobulin E; STAT3, Signal transducer and activator of transcription 3; FOXP3, Forkhead Box Protein P3; TGF-β1, transforming growth factor-β1; VEGF, vascular endothelial growth factor; ERK, extracellular signal-regulated kinase; CCL, c-c motif chemokine ligand; TNF-α, tumor necrosis factor-alpha; IκB, Inhibitor of kappaB; CCR, C-C-Motif Receptor; LPS, lipopolysaccharide; HSP72, heat shock protein 72; JNK, c-Jun N-terminal kinase; COPD, chronic obstructive pulmonary disease; SD, sprague dawley; NF-κB, nuclear factor-kappaB; PAI-1, Plasminogen activator inhibitor-1; HDAC2, histone deacetylase 2; PAH, pulmonary arterial hypertension; MCT, monocrotaline; AKT, protein kinase B; NOS, nitric oxide synthase; ET-1, Endothelin-1; ICAM-1, intercellular cell adhesion molecule-1; α-SMA, alpha-smooth muscle actin; VCAM, vascular cell adhesion molecule; Smad, small mothers against decapentaplegic; MLE-12, mouse lung epithelial-12; ADAMTS, A Disintegrin and Metalloproteinase with Thrombospondin Motifs; ID1, inhibitor of DNA, binding 1; BMPR2, bone morphogenetic protein receptor 2; A_2A_R, A2A adenosine receptor; CXCR4, C-X-C chemokine receptor 4; SDF-1, stromal derived factor-1; MMP-9, matrix metalloproteinase-9; Hyp, hydroxyproline; MDA, malondialdehyde; TGF-β1, transforming growth factor-β1; i.p., intraperitoneal; i.n., intranasal; Treg, regulatory T-cell; i.g., intragastric; IκB-α, Inhibitor of kappaB-α; MAPK, Mitogen-activated protein kinase; Refs., reference.

### 5.4 Chronic obstructive pulmonary disease

COPD is characterized by airway remodeling and progressive lung inflammation, which are the main features of an increase in the number of alveolar macrophages, neutrophils, and T lymphocytes (Barnes, 2014; Chang et al., 2014). Cigarette smoking is one of the primary causes of COPD, resulting in 5 million deaths annually (Jones et al., 2017; Labaki and Rosenberg, 2020).

Hao et al. (2021) utilized cigarette smoke extract-induced mouse lung epithelial cell model and cigarette smoke and LPS-induced mouse model to investigate the anti-inflammatory effect of BA in COPD. They found that BA (25, 50, and 100 mg/kg, i.g. administration) inhibited inflammatory cell infiltration and the secretion of pro-inflammatory factors by regulating the heat shock protein 72 (HSP72)-mediated c-Jun N-terminal kinases (JNK) pathway. In addition, Zhang *et al.* showed that BA (i.g. administration) dosages ranging from 40 to 160 mg/kg suppressed the expression of inflammatory factors in cigarette smoke (extract)-induced human bronchial epithelial cells and airway tissues through suppressing the inflammatory response, enhancing histone deacetylase 2 protein expression, and inhibiting the activation of NF-κB and its downstream target of PAI-1 (Zhang et al., 2021). Using the same model, a study by Zeng *et al.* indicated that BA (20, 40, and 80 mg/kg, i.g. administration) reduced cigarette smoke-induced inflammation in human type II pneumocytes and rats by suppressing IL-6, IL-8, and TNF-α *via* the inhibition of NF-κB activation (Lixuan et al., 2010).

According to recent advances, the mechanism of COPD mitigation by BA revolves around inhibition of the inflammatory response, mainly through tight regulation of the HSP72-mediated JNK pathway, HDAC2/NF-κB/PAI-1 pathway, and NF-κB pathway (Table 4).

### 5.5 Pulmonary arterial hypertension

PAH is a progressive and fatal disease with rapid onset and mortality in 7 people for every 100 person-years in Asian countries (Chung et al., 2015). Pulmonary endothelial cell dysfunction, pulmonary artery smooth muscle proliferation, and right ventricular hypertrophy are all involved in the development and formation of PAH (Xu et al., 2022b).

In a study by Luan et al. (2015), the rats with monocrotaline (MCT)-induced PAH were administered BA (i.g. administration) at a daily dose of 100 mg/kg. The results showed that BA downregulated the mRNA levels of TNF-α, IL-13, IL-6, and ET-1 in pulmonary tissues *via* blocking the activation of the NF-κB signaling pathway to attenuate PAH. Utilizing the same model, Xue et al. (2021) found that BA (20, 50, and 200 mg/kg, i.g. administration) profoundly reduced the mRNA and protein levels of inflammatory factors, excessive proliferation, migration of pulmonary artery smooth muscle cells, and vascular remodeling *via* the TNF‐α/bone morphogenetic protein receptor 2 (BMPR2) signaling pathway. In an MCT-induced rat model, BA (20, 100, and 200 mg/kg, i.p. injection) protected the rats from the severity of pulmonary vascular remodeling and cardiorespiratory injury *via* AKT/ERK/NF-κB signaling pathways and p-eNOS protein (Yan et al., 2019). In another PAH model, the relief of chronic hypoxia-induced PAH by BA (60 mg/kg, i.p. injection) through the AKT pathway was confirmed. In addition, the results of *in vivo* studies suggested that BA performed a therapeutic role *via* the attenuation of the adenosine A_2A_ receptor-induced SDF-1/CXCR4/PI3K/AKT pathway (Huang et al., 2017). Yan et al. (2016) demonstrated that 30 mg/kg of BA (i.p. injection) relieved the hypoxia-induced PAH model by mitigating pulmonary hypertension, pulmonary arteriole, and right ventricular remodeling and ameliorating hypoxic cor pulmonale by suppressing the p38 MAPK pathway and the expression of matrix metalloprotein (MMP)-9.

In conclusion, the suppression of inflammatory factors, the TNF‐α/BMPR2 pathway, the SDF-1/CXCR4/PI3K/AKT pathway, and the p38 MAPK pathway seem to be essential to the regulation of PAH by BA (Table 4).

### 5.6 Pulmonary fibrosis

PF is a chronic, progressive, and deadly lung disease primarily affecting middle-aged and older people (Sharif, 2017). It is generally accepted that the occurrence of PF is abnormal wound healing after repeated alveolar injury in genetically susceptible individuals, leading to the destruction of the lung parenchyma, and deposition of extracellular matrix in fibroblasts and alveolar epithelium. In addition, improperly activated alveolar epithelial cells (AECs) and fibroblasts cause the fibrotic response (King et al., 2011; Ptasinski et al., 2021).

A recent study indicated that 50 mg/kg BA (i.p. administration) effectively suppresses bleomycin (BLM)-induced PF in rats by decreasing the expression of hydroxyproline, TGF-β, collagen I, collagen III, and TNF-α, enhancing the function of anti-oxidative stress, and inhibiting apoptotic protein expression. Moreover, this study also showed that 80 μg/mL BA reverses BLM-induced fibroblast proliferation, regulating the expression of phosphoinositide 3-kinase (PI3K)/AKT and calcium/calmodulin-dependent kinase II *in vitro* (Zhao et al., 2020). Another *in vivo* study revealed that 120 mg/kg BA (i.p. administration) was found to block the TGF-β1-induced ERK1/2 signaling pathway *via* the adenosine A_2A_ receptor, leading to the alleviation of fibrosis caused by BLM (Huang et al., 2016). In recent years, the targeted omics approach for biomarkers discovery has been an effective approach for evaluating the effectiveness and mechanisms of BA (Ullah et al., 2022). In the study of Chang et al. (2021), the researchers administered 25–100 mg/kg BA to rat models with PF caused by BLM. The results demonstrated that BA ameliorated the levels of MDA and SOD to relieve oxidative stress. Furthermore, BA was identified to potentially alleviate PF by regulating four vital metabolic markers, including taurine, hypotaurine, glutathione, and glycerophospholipid metabolism.

TGF-β acts as a potent driver of PF progression by induction of epithelial-mesenchymal transition (EMT) (Qian et al., 2018). At doses of 2–50 μM, BA appears to be implicated in the radiation-induced EMT of AECs by inhibiting TGF-β and ERK/GSK signaling pathways mediated fibrosis (Lu et al., 2017). In terms of immunoregulatory mechanisms, i.p. injection of 100 mg/kg BA decreased PF in mouse models caused by silica by altering the Th17/Treg responses by lowering Th17 cells, activating Treg cells, and inhibiting IL-6 and IL-23 (Liu et al., 2015).

In summary, BA alleviates PF through a variety of mechanisms, including inhibition of the inflammatory response, oxidative stress, EMT, apoptosis and immune modulation (Table 5).

**TABLE 5:** Summary of the targets/pathways/mechanisms and effects of baicalin on pulmonary fibrosis and lung cancer.

Disease	Inducer	Experimental model	Dose	Targets/pathways/mechanisms	Effects	Refs.
PF	*In vivo*: BLM (5 mg/kg, i.t. instillation)	*In vivo*: adult female Wistar rats	*In vivo*: 50 mg/kg (i.p. administration)	*In vivo*: Hyp, collagen I, collagen III, TGF-β, TNF-α, MDA, caspase-3, Bax, cells in the BALF↓	Inhibits pulmonary fibrosis, inflammation, tissue apoptosis, collagen deposition, oxidative stress-associated damage, and proliferation and arrests cell cycle *via* the inhibition of the CaMKⅡ and AKT signaling pathways	Zhao et al. (2020)
				GSH-px, T-SOD, GSH, Bcl-2↑		
	*In vitro*: BLM (20 μg/mL)	*In vitro*: rat primary lung fibroblasts	*In vitro*: 80 μg/mL	*In vitro*: cyclin A, cyclin D, cyclin E, PCNA, p-AKT/t-AKT, p-CaMKII/t-CaMKII↓		
PF	*In vivo*: BLM (5 U/kg, i.t. injection)	*In vivo*: wild type Balb/c mice and A2aR homozygous knockout mice	*In vivo*: 120 mg/kg (i.p. injection)	*In vivo*: Hyp, TGF-β1, p-ERK/t-ERK, p-ERK1/2↓	Attenuates pulmonary fibrosis *via* adenosine A2aR-related TGF-β1-induced ERK1/2 signaling pathway	Huang et al. (2016)
				A2aR↑		
				ERK1/2→		
PF	*In vivo*: BLM (5 mg/kg, i.t. instillation)	*In vivo*: male SD rats	*In vivo*: 25 and 100 mg/kg (gavage)	*In vivo*: Hyp, MDA↓	Improves pulmonary fibrosis by the regulation of four key biomarkers involving taurine and hypotaurine metabolism, glutathione metabolism and glycerophospholipid metabolism	Chang et al. (2021)
				SOD↑		
				Regulates taurine and hypotaurine metabolism, glutathione metabolism, and glycerophospholipid metabolism		
PF	*In vitro*: radiation (8 Gy of^60^Co γ-rays at a dose rate of 3.64 Gy/min)	*In vitro:* primary rat type II AEC	*In vitro:* 2, 10, and 50 μΜ	*In vitro:* vimentin, snail, α-SMA, TGF-β, p-ERK/ERK, p-GSK3β/GSK3β, p-Smad2/Smad2, p-Smad3/Smad3↓	Alleviates EMT transition *via* TGF-β and ERK/GSK3β signaling pathways	Lu et al. (2017)
				E-cadherin↑		
PF	*In vivo*: silicosis (2.5 per mouse, oral-tracheal instillation)	*In vivo*: female C57BL/6 mice	*In vivo*: 2 mg per mouse (i.p. administration)	*In vivo*: total cells in BALF, neutrophils number, lymphocytes number, macrophages number, the percentage of CD4+IL-17A+T cells, TNF-α, IL-4↓	Inhibits the Th17 response and reduces inflammation and fibrosis	Liu et al. (2015)
				The percentage of CD4+Foxp3+T-cell↑		
				The percentage of CD4+T-cell co-expressed IFN-γ↓		
				mRNA and protein expression of IL-6↓		
				mRNA expression of Th17A, RORγt, GATA-3, TGF-β, IFN-γ, and IL-23↓		
				mRNA expression of IL-10 and Foxp3↑		
LC	*In vivo*: implants 5 × 10^6^/mL tumor cell	*In vivo*: female BALB/c-nu mice	*In vivo*: 20 mg/kg	*In vivo*: p-Akt, p-mTOR, CDK4, and cyclin E2↓	Exerts antitumor activity *via* inducing Akt-dependent cell cycle arrest and promoting apoptosis	Sui et al. (2020)
	*In vitro*: NA	*In vitro*: H1650 and H1299 cells	*In vitro*: 0–150 μg/mL	*In vitro*: Bax, cleaved caspase-9↑		
				Bcl-2, CDK2, CDK4, cyclin E2, p- Akt, p- mTOR↓		
LC	*In vivo*: infects with 1 × 10^6^ NCI-H460 cells	*In vivo*: BALB/c nude mice	*In vivo*: 40 and 60 mg/kg (oral gavage)	*In vivo*: vimentin, p-PDK1, p-AKT↓	Impedes EMT by inhibiting the PDK1/AKT pathway	Chen et al. (2021)
				E-cadherin↑		
	*In vitro*: NA	*In vitro*: A549, NCI-H460, and BEAS-2B cells	*In vitro*: 0, 15, and 30 μM	*In vitro*: vimentin, p-PDK1, p-AKT↓		
				E-cadherin↑		
LC	*In vitro*: NA	*In vitro*: A549 and H1299 cells	*In vitro*: 60 μg/mL	*In vitro*: miR-340-5p↑	Elicits antitumor activities by affecting the miR-340-5p/NET1 axis	Zhao et al. (2021)
				mRNA and protein expression of NET1↓		
LC	*In vitro*: NA	*In vitro*: A549 and H1299 cells	*In vitro*: 0–80 μmol/L	*In vitro*: SIRT1, p-AMPK/AMPK, c-caspase-3↑	Inhibits the proliferation and migration by activating the SIRT1/AMPK signaling pathway	You et al. (2018)
				p-mTOR/mTOR, active MMP-2, active MMP-9↓		
LC	*In vivo*: athymic nude mice injected with H441 lung cancer cell (4 ×10^6^/0.1 mL)	*In vivo*: athymic nude mice	*In vivo*: 20 and 50 mg/kg (i.p. injection)	*In vivo*: p-Histones H3, p-ERKs, PBK/TOPK↓	Inhibits the proliferation of lung cancer *via* decreasing the PBK/TOPK downstream signaling molecules Histone H3 and ERK2	Diao et al. (2019)
	*In vitro*: NA	*In vitro*: JB6 Cl41 cells, H441, H1975, H1299, and A549 cells	*In vitro*: 25, 50, and 100 μM	*In vitro*: p-Histones H3/Histones H3, p-EKRs, p-ERKs/ERKs, PBK/TOPK↓		
LC	*In vitro*: NA	*In vitro*: A549 cells and A549/DDP cells (DDP-resistant human lung cancer cells)	*In vitro*: 1–8 μg/mL	*In vitro*: MAPK2 mRNA and protein↓	Inhibits proliferation and invasion and attenuates DDP resistance by decreasing protein expression of MAPK2 and p-Akt	Xu et al. (2017b)
				p-AKT↓		
				AKT→		
LC	*In vitro*: NA	*In vitro*: A549 and H2009 cells	*In vitro*: 75 μM	*In vitro*: p-p38/p38, ROS, cleaved-PARP↑	Enhances the anticancer activity of TRAIL *via* p38 activation and ROS accumulation	Zhang et al. (2017)

Hyp, hydroxyproline; TGF-β1, transforming growth factor-β1; TNF-α, tumor necrosis factor-α; MDA, malondialdehyde; Bax, Bcl-2-associated X protein; BALF, bronchoalveolar lavage fluid; GSH, glutathione; GSH-px, glutathione peroxidase; SOD, superoxide dismutase; Bcl-2, B-cell lymphoma-2; AKT, protein kinase B; CaMKII, calmodulin-dependent kinase II; ERK, extracellular signal-regulated kinase; α-SMA, alpha-smooth muscle actin; GSK3β, glycogen synthase kinase-3, beta; Smad, small mothers against decapentaplegic; EMT, epithelial-mesenchymal transition; AEC, alveolar type epithelial cell; Foxp3, Forkhead Box P3; IFN-γ, interferon-γ; RORγt, retinoid-related orphan nuclear receptor γt; GATA-3, GATA-binding protein 3; mTOR, mechanistic target of rapamycin; CDK, cyclin-dependent kinase; PDK1, phosphoinositide-dependent protein kinase 1; NET1, neuroepithelial cell transforming 1; SIRT1, sirtuin 1; AMPK, adenosine monophosphate-activated protein kinase; SD, Sprague–Dawley; MMP, matrix metalloproteinase; MAPK, Mitogen-activated protein kinase; DDP, cisplatin; ROS, reactive oxygen species; PARP, poly ADP-ribose polymerase; BLM, bleomycin; i.t., intratracheal; PCNA, proliferating cell nuclear antigen; IL, interleukin; MMP, matrix metalloprotein; PBK/TOPK: PDZ-binding kinase/T-LAK, cell-originated protein kinase; A2aR: adenosine A2a receptor; Refs., reference; PF, pulmonary fibrosis; TRAIL, tumor necrosis factor-related apoptosis-inducing ligand; NA, not applicable.

### 5.7 Lung cancer

LC remains the primary cause of cancer deaths in the world (Wei et al., 2015; Saman et al., 2022). As the most common subtype of LC, non-small cell lung cancer (NSCLC) accounts for around 85% of LC cases (Fiero et al., 2019). Currently, surgery, chemotherapy, radiation, and targeted molecular treatment are the main treatment options for LC patients (South et al., 2020). However, the side effects and toxicity of these techniques have become limiting factors in the clinical treatment of lung cancer (Luan et al., 2015; Yamamoto et al., 2022).

Sui et al. (2020) found that 20 mg/kg of BA can trigger apoptosis and block cell cycle G1/S transition in carcinoma of the lung cells *via* the AKT/mechanistic target of rapamycin (mTOR) pathway. NSCLC is highly prone to invasion and metastasis, which are closely associated with the occurrence of EMT (Xia et al., 2014). In this regard, Chen reported that BA inhibited the EMT of NSCLC through the PDK1/AKT pathway (Chen et al., 2021). Recently, the importance of miRNAs in cancer progression has been reported (He et al., 2005; Lee and Dutta, 2007). Zhao et al. (2021) discovered that BA (60 μg/mL) restrained cell proliferation and invasion, promoted apoptosis, and arrested the G1/S phase *via* the miR-340-5p/NET1 axis, exerting anti-lung cancer effects. In an *in vitro* study, BA (20–80 μmol/L) inhibited cell viability, triggered apoptosis, and decreased cell migration and invasion by inhibiting the activation of the sirtuin 1 (SIRT1)/adenosine monophosphate-activated protein kinase (AMPK) signaling pathway (You et al., 2018). In several studies, overexpression of PDZ-binding kinase/T-LAK cell-originated protein kinase (PBK/TOPK) has been related to a poor prognosis and is a crucial target for anticancer drugs (Lei et al., 2013; Han et al., 2021). Diao et al. (2019) found that BA inhibited PBK/TOPK activities by directly binding with PBK/TOPK *in vitro* and *in vivo*.

Despite the successful efficacy of chemotherapy, there is growing concerned about chemotherapeutic agents developing resistance (Siddik, 2003; Apps et al., 2015; Pan et al., 2019). Considering this, researchers found that combining BA and DDP (cisplatin) inhibited tumor cells significantly more than DDP or BA alone in A549 and A549/DDP cells (DDP-resistant cells). The attenuation of the resistance of DDP was connected with downregulating MARK2 and p-Akt proteins (Xu et al., 2017b). In addition, tumor necrosis factor-related apoptosis-inducing ligand (TRAIL) is a promising target for overcoming cancer cell resistance (von Karstedt et al., 2017). BA (75 μM) can increase TRAIL-induced apoptosis and intracellular reactive oxygen species generation in cancer cells *via* activating p38 MAPK, enhancing the antitumor effectiveness of TRAIL (Zhang et al., 2017). The findings of the current investigations suggested that targeting MAPK is a significant pathway to reduce drug resistance in cancer. The AKT/mTOR, the PDK1/AKT, the SIRT1/AMPK pathways, and the regulation of active MMP-2, active MMP-9, vimentin, and E-cadherin are connected with the relief of LC (Table 5).

## 6 Discussion and outlook

**FIGURE 2:**
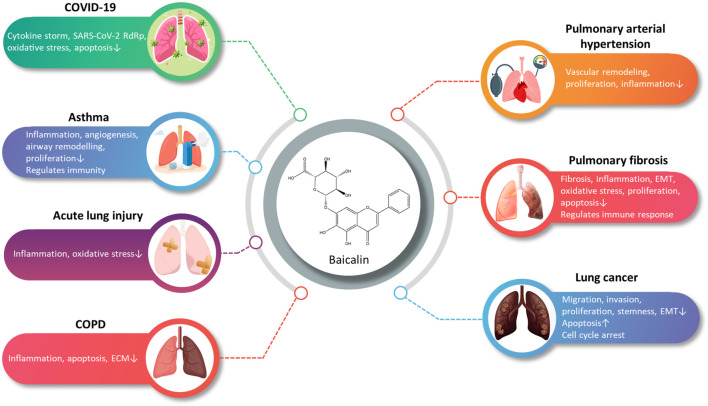
A summary of the main pharmacological effects of baicalin and its mechanisms involved in respiratory diseases (graphics courtesy of freepik.com).

Through this review, we have attempted to summarize a comprehensive review of research advances for BA in pharmacokinetics, strategies to improve bioavailability, and the treatment of disorders related to the respiratory system. Despite the poor bioavailability and solubility of BA, the development of DDSs for BA has improved the bioavailability, pulmonary targeting, and solubility, thereby improving the efficacy. The multiple pharmacological activities of BA suggest a broad prospect for the prevention and treatment of ALI, PF, COVID-19, LC, PAH, COPD, and asthma (Figure 2). At present, studies on BA are still in the preclinical stage. This review provides a valuable reference for subsequent pharmacological studies and clinical applications of BA.

**FIGURE 3:**
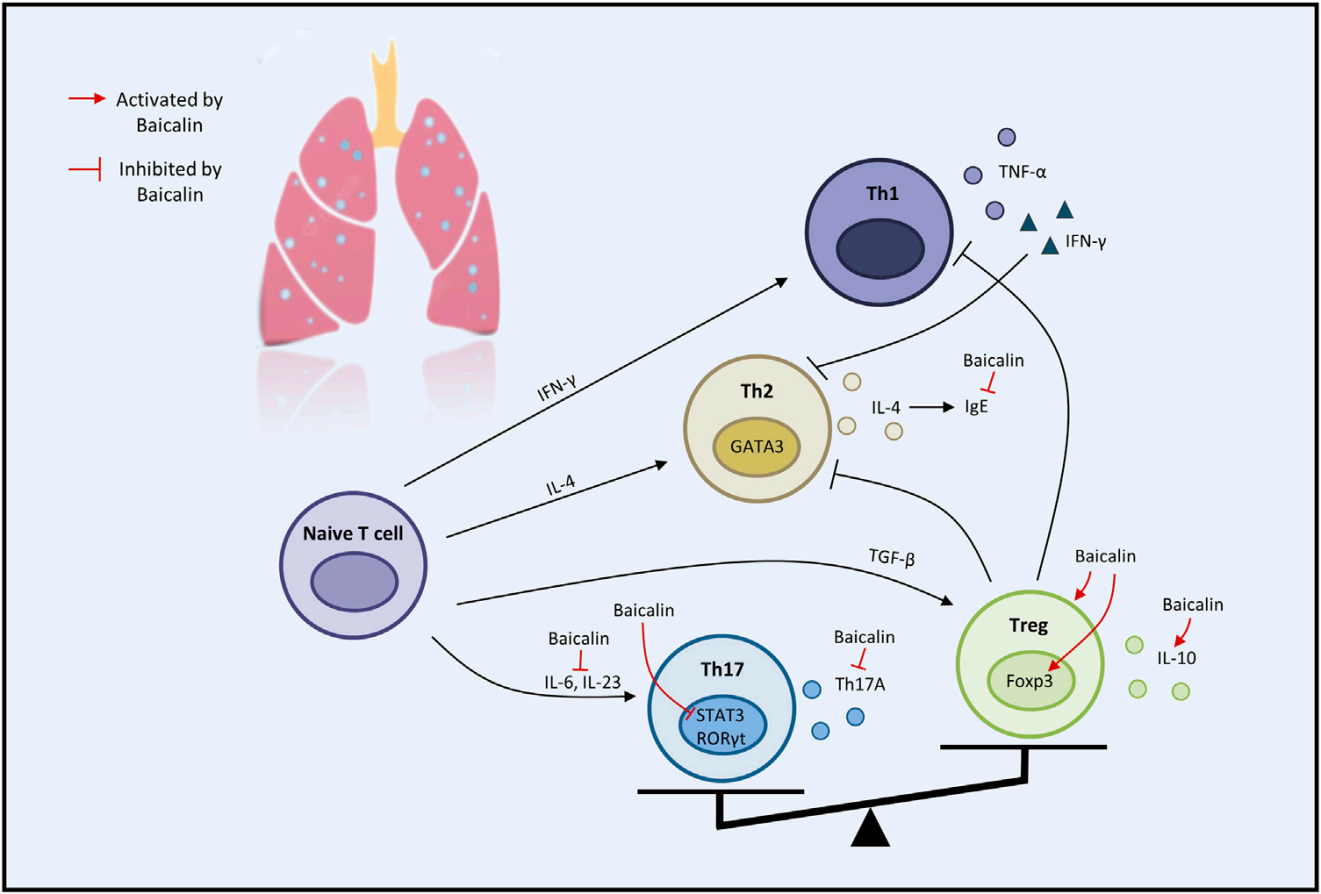
The therapeutic effects of baicalin on immune modulation on respiratory disease (graphics courtesy of freepik.com).

**FIGURE 4:**
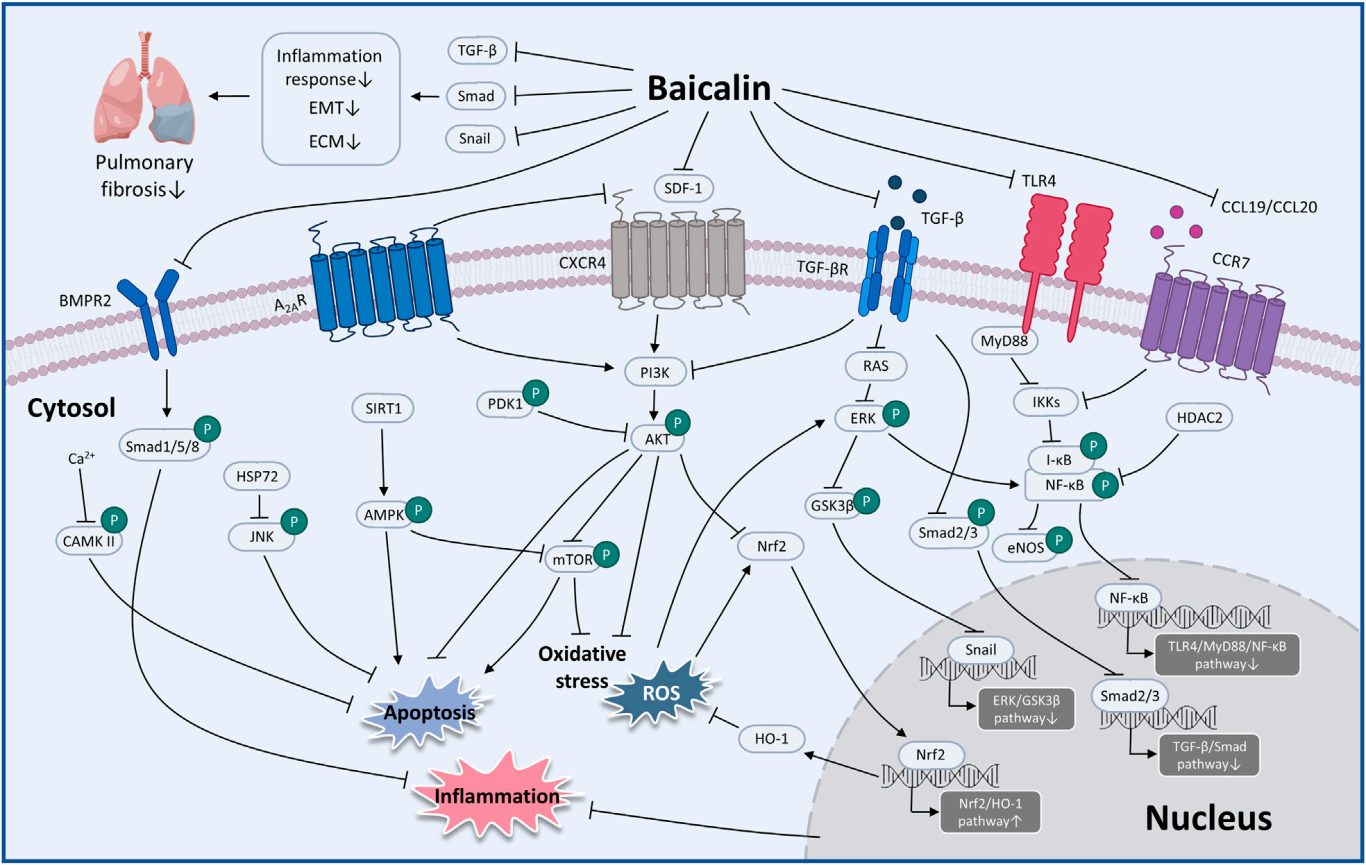
The therapeutic mechanism and critical pathways of baicalin against respiratory diseases (graphics courtesy of figdraw.com/static/index.html#/ and freepik.com).

Considerable preclinical studies have shown that BA has excellent therapeutic potential to treat respiratory diseases in both *in vivo* and *in vitro* settings. BA exerts therapeutic effects mainly through mediating upstream oxidative stress, inflammation, apoptosis, and immune response pathways. In the regulation of oxidative stress, the PI3K/AKT/eNOS pathway and the Nrf2-mediated HO-1 pathway are critical factors associated with the protective effects of BA on COVID-19 and ALI. Regarding the inflammatory response, TNF-α, IL-1β, IL-6, GM-CSF, IL-8, IL-4, and IL-10 are involved in BA to alleviate COVID-19, ALI, PF, COPD, PAH, and asthma. Regarding the regulation of apoptosis molecules, caspase-3, caspase-9, Bcl-2, and Bax are essential factors in alleviating LC, PF, and COVID-19. Immune modulation therapy has sparked the interest of researchers. The underlying mechanisms of BA in the treatment of immune modulation on PF and asthma manifest as the regulation of Th17/Treg responses by promoting Treg cell differentiation, inhibiting the expression of Th17A, and reducing the levels of IL-6, IFN-γ, IL-4, and IL-23 (Figure 3). Although the target of BA for the treatment of respiratory diseases is still inconclusive. Phosphodiesterase 4 (PDE4) might be a potential therapeutic target for treating respiratory diseases. And PDE4 already has targeted inhibitors approved for treating severe COPD, such as roflumilast (Crocetti et al., 2022; Herrmann et al., 2022). In addition, GSK-256066, a highly selective inhaled PDE4 inhibitor, has been in phase II clinical trials for asthma and COPD (Pagès et al., 2009). BA was found to selectively inhibit the enzymatic activity of PDE4A and 4B to relieve allergic asthma (Park et al., 2016). Thus, it indicates that PDE4 may be a candidate target for BA to target respiratory diseases, which requires further experimental proof. Importantly, we discovered that BA exhibits anti-cancer effects *via* inducing apoptosis and oxidative stress, which is the opposite of BA for the treatment of PF. It suggests that BA exerts a bidirectional regulatory effect to protect the lung. The multiple pharmacological activities suggest that BA is a promising compound for the prevention and treatment of ALI, PF, COVID-19, LC, PAH, COPD, and asthma (Figure 4).

This review identifies a plausible mechanism for the therapeutic efficacy of BA in treating respiratory diseases. However, the experiments have almost always verified the conclusion *in vivo* and *in vitro*. More clinical evidence is needed to verify efficacy. Several aspects of BA need to be investigated before its clinical application. Firstly, the poor water solubility of BA leads to low oral bioavailability. Researchers have developed strategies to improve the bioavailability of BA, including liposomes, nano-emulsions, micelles, phospholipid complexes, solid dispersions, and inclusion complexes (Li J. et al., 2009; Li et al., 2013; Yue et al., 2013; Zhao et al., 2013; Wu et al., 2014; Zhang et al., 2014). Modified versions of these different formulations of BA would be carried out in animal experiments to find their optimal effective dose. And there is also the issue of acute and long-term toxicity of BA in animal models of different respiratory diseases. Secondly, therapeutic doses of BA varied widely across studies. This may be due to disease type, disease state, route of administration, time point of administration (prophylactic or therapeutic administration), number of days of treatment, age, weight, sex of the animals, and environmental factors, etc. Thus, when BA is used as a treatment for respiratory diseases, it needs to be tailored to the specific situation. In addition, as a common dosage form for the treatment of respiratory diseases, inhalation of BA has not been studied. BA is reported to be insoluble in water that may be the main reason for limiting its candidate for the development of inhalation preparation. A number of new formulations have been developed to improve water solubility, including inclusion complexes and phospholipid complexes, etc. BA has shown improved properties as an inhalant component of chinese herbal compound. Transnasal aerosol inhalation of Tanreqing (containing BA) increased the drug concentration of BA in the lungs and showed better anti-inflammatory effects on LPS-induced ALI in mice, compared to intravenous administration (Chen T. F. et al., 2022). This indicates the potential of BA as an inhalation. Finally, with the advancement of multi-omics studies, those approaches can be used to explore the mechanism of the effects of BA in the treatment of respiratory diseases and to gain a clearer understanding of the multi-target regulatory network of BA. In general, this review presents novel perspectives on the pharmacological effects of BA on respiratory diseases. It must be acknowledged that this review also has some limitations, including the source of BA, the experimental method, and the choice of dose.

## Glossary

ACE2: angiotensin-converting enzyme-2
AECs: alveolar epithelial cells
ALI: acute lung injury
AMPK: adenosine monophosphate-activated protein kinase
Ang II: angiotensin II
AUC: plasma drug concentration-time curve
BA: Baicalin
BE: Baicalein
BLM: bleomycin
BMPR2: bone morphogenetic protein receptor 2
CAT: catalase
CCR: CC-chemokine receptor
CCL: C-C chemokine ligand
C_max_: maximum serum concentration
COPD: chronic obstructive pulmonary disease
COVID-19: coronary viral disease-2019
CS: cytokine storm
DDP: cisplatin
DDSs: drug delivery systems
EMT: Epithelial-mesenchymal transition
ERK: extracellular signal-regulated kinase
GM-CSF: Granulocyte-macrophage colony stimulating factor
HO-1: heme oxygenase-1
HSP72: heat shock protein 72
i.g.: intragastric gavage
i.p.: intraperitoneal
IgE: immunoglobulin E
IL: interleukin
JNK: c-Jun N-terminal kinases
L-BA: BA liposome
LC: lung cancer
LPS: lipopolysaccharide
MAPK: mitogen-activated protein kinase
MCN: mesoporous carbon nanopowder
MCT: monocrotaline
MDA: malondialdehyde
MMP: matrix metalloprotein
mTOR: mechanistic target of rapamycin
MyD88: myeloid differentiation factor 88
NF-κB: nuclear factor-kappa B
NLRP3: nod-like receptor pyrin containing 3
Nrf2: nuclear erythroid factor 2
NSCLC: Non-small cell lung cancer
OVA: ovalbumin
PAH: pulmonary arterial hypertension
PAI-1: plasminogen activator inhibitor-1
PBK/TOPK: PDZ-binding kinase/T-LAK cell-originated protein kinase
PDE4: phosphodiesterase 4
PF: pulmonary fibrosis
PI3K: Phosphoinositide 3-kinase
SARS-CoV-2: severe acute respiratory syndrome coronavirus 2
SBG: Scutellaria baicalensis Georgi
SEDDS: self-emulsifying drug delivery systems
SIRT1: sirtuin 1
SOD: superoxide dismutase
TCM: traditional Chinese medicine
TGF-β: transforming growth factor-β
TLR4: Toll Like Receptor 4
TNF-α: tumor necrosis factor-α
TRAIL: Tumor necrosis factor-related apoptosis-inducing ligand
UGT: UDP-glucuronosyltransferase
VEGF: vascular endothelial growth factor
β-CD: β-cyclodextrin
